# Downregulation of miR-503 Promotes ESCC Cell Proliferation, Migration, and Invasion by Targeting *Cyclin D1*

**DOI:** 10.1016/j.gpb.2017.04.003

**Published:** 2017-06-09

**Authors:** Lanfang Jiang, Zitong Zhao, Leilei Zheng, Liyan Xue, Qimin Zhan, Yongmei Song

**Affiliations:** State Key Laboratory of Molecular Oncology, National Cancer Center/Cancer Hospital, Chinese Academy of Medical Sciences and Peking Union Medical College, Beijing 100021, China

**Keywords:** Esophageal squamous cell carcinoma, miR-503, *Cyclin D1*, Proliferation, Migration and invasion

## Abstract

**Esophageal squamous cell carcinoma** (ESCC) is one of the most aggressive cancers in China, but the underlying molecular mechanism of ESCC is still unclear. Involvement of microRNAs has been demonstrated in cancer initiation and progression. Despite the reported function of **miR-503** in several human cancers, its detailed anti-oncogenic role and clinical significance in ESCC remain undefined. In this study, we examined miR-503 expression by qPCR and found the downregulation of miR-503 expression in ESCC tissue relative to adjacent normal tissues. Further investigation in the effect of miR-503 on ESCC cell **proliferation**, migration, and invasion showed that enhanced expression of miR-503 inhibited ESCC aggressive phenotype and overexpression of *CCND1* reversed the effect of miR-503-mediated ESCC cell aggressive phenotype. Our study further identified *CCND1* as the target gene of miR-503. Thus, miR-503 functions as a tumor suppressor and has an important role in ESCC by targeting *CCND1*.

## Introduction

Esophageal cancer (EC) is the fourth leading cause of cancer-related death in China, and esophageal squamous cell carcinoma (ESCC) accounts for 90% of EC [1], [2]. Despite the great efforts in the development of preventive approaches, diagnostic technologies, and treatment modalities made for ESCC, the five-year survival rate of ESCC is below 20% and the exact molecular mechanism and pathological process of ESCC remain poorly elucidated [3]. Therefore, understanding the underlying molecular mechanisms might provide novel insights into the prevention and treatment of ESCC.

MicroRNAs (miRNAs) are non-coding RNAs, which can bind to their target protein-coding genes at their 3′UTRs based on sequence complementarity, consequently affecting the mRNA stability or interfering with protein translation [4], [5], [6], [7], [8]. miRNAs play crucial roles in carcinogenesis and cancer development, and a growing body of evidence has shown that many miRNAs are aberrantly expressed and dysfunctional in ESCC [9], [10], [11]. For instance, miR-425 expression is significantly upregulated in ESCC [12]. miR-377 suppresses ESCC initiation and development by targeting *VEGF* and *CD133* [13]. The deregulation of miR-503 has been reported in various human cancers [14], [15], [16], [17], [18]. miR-503 can repress epithelial-mesenchymal transition (EMT) and inhibit metastasis of osteosarcoma by targeting *c-myb* [14]. It can also inhibit glioma cell proliferation and invasion by targeting *L1CAM* encoding L1 cell adhesion molecule [15]. *CCND1*, which encodes cyclin D1, is the target of miR-503 in breast cancer cells and endometrioid endometrial cancer [16], [17]. A significant elevation in miR-503 expression has been reported in the human ESCC cell line EC9706 compared to the normal human esophageal epithelial cell (HEEC), whereas downregulated miR-503 expression suppresses proliferation, migration, and invasion in EC9706 cells [18]. However, the expression of miR-503 in other ESCC cell lines and ESCC tissues has not been reported, and whether miR-503 has the tumor suppressive role in ESCC remains to be addressed.

In this study, we demonstrated that the expression of miR-503 was suppressed in ESCC tissue, whereas overexpression of miR-503 inhibited aggressive phenotypes of ESCC cells *in vitro*. Cyclin D1 is an important protein during the development of ESCC, and *CCND1* was demonstrated to be the target of miR-503. Our findings provide a mechanism by which miR-503 mediates ESCC malignant phenotype formation.

## Results

### miR-503 expression is downregulated in human ESCC tissue samples and cell lines

**Figure 1:**
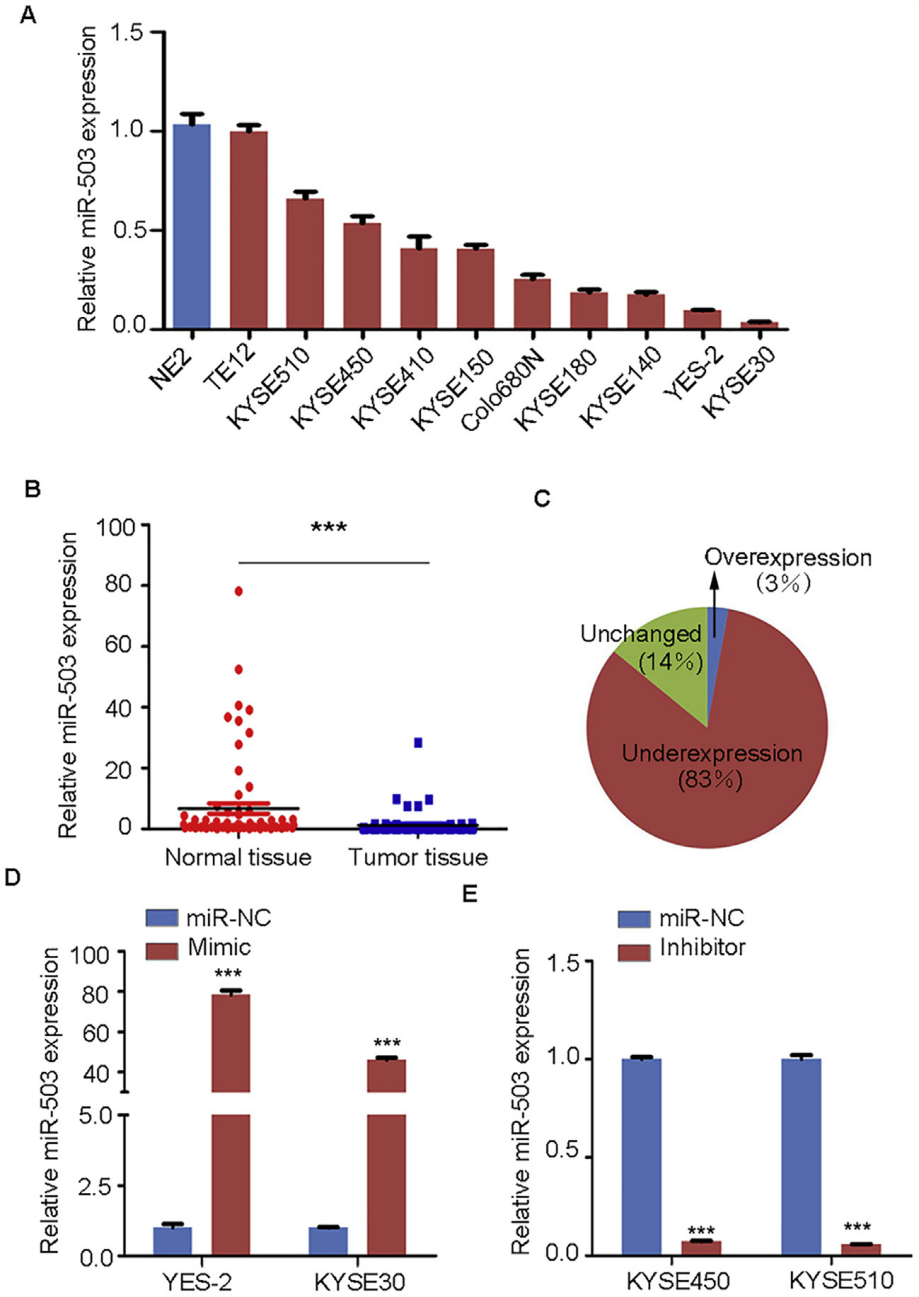
**miR-503 expression was down-regulated in ESCC tissues and cell lines** **A.** miR-503 expression in NE2 and 10 ESCC cell lines was evaluated using qPCR and presented as fold change relative to the expression in NE2. **B.** Relative expression of miR-503 was evaluated using qPCR in 71 pairs of ESCC samples and the corresponding adjacent non-cancerous samples. **C.** Down-regulation of miR-503 expression was observed in 83% of ESCC tumor samples compared to the corresponding adjacent non-cancerous tissues. **D.** Overexpression of miR-503 mimic in KYSE30 and YES-2 cell lines was analyzed using qPCR. **E.** Silencing of miR-503 expression after transfecting the inhibitor in KYSE450 and KYSE510 cell lines was analyzed using qPCR. ^*^P < 0.05, ^**^P < 0.01, ^***^P < 0.001, *t*-test. All the experiments were performed at least three times. *U6* was used as the internal control for miR-503 expression.

Many ESCC cell lines, such as KYSE30 and KYSE450, have been employed for ESCC studies and each cell line may reflect a different aspect of ESCC features [3]. We thus examined the endogenous expression of miR-503 in 10 commonly-used ESCC cell lines, using the immortalized esophageal epithelial cell line NE2 as a control. qPCR analysis showed that compared to the NE2 cell line, miR-503 expression was markedly decreased in most of the ESCC cell lines examined (Figure1A).

Currently, there is no report on the expression of miR-503 in ESCC tissues. We next used qPCR to evaluate miR-503 expression in 71 pairs of tumor samples and the adjacent non-tumorous tissue samples from ESCC patients. Our data showed that compared to the adjacent non-tumorous tissues from the same patient, there is a significant decrease in miR503 expression in the tumor samples from 83% of the ESCC patients (*P* *=* 0.0021, Figure1B and C).

To evaluate the possible association between miR-503 expression and clinicopathological characteristics of ESCC patients, we then analyzed the miR-503 expression levels regarding the clinicopathological information of ESCC patients using Pearson’s *χ*^2^ test. However, we did not find significant association between miR-503 expression and gender, age, or lymph node metastasis (Table 1).

**Table 1:** **Clinical characteristics of the ESCC patients and miR-503 expression**

**Characteristics**	**Total (*n* = 71)**	**miR-503 expression**		***P***
		**High**	**Low**	
*Age*				
≥60	20	11	9	0.656
<60	51	25	26	

*Gender*				
Male	48	24	24	0.866
Female	23	12	11	

*Lymph node metastasis*				
Yes	26	13	13	0.928
No	45	23	22	

*Note*: Association between miR-503 expression and various clinicopathological parameters was determined using a Pearson’s chi-squared (*χ*^2^) test. *P* < 0.05 indicates significant difference. Expression level of miR-503 was categorized into 'high' and 'low' using the median value as the cutoff point [13].

### miR-503 negatively regulates proliferation, migration, and invasion and could induce G1/S arrest of ESCC cells

Downregulation of miR-503 expression in ESCC cell lines and tissues relative to normal human esophageal epithelial cell and adjacent normal tissues indicates miR-503 might be a negative regulator of tumorigenesis in ESCC. To explore the function of miR-503 in ESCC, a transient transfection strategy was employed to perform gain-of-function and loss-of-function assays. YES-2 and KYSE30 cell lines, which possessed low expression level of miR-503, were chosen to transfect the mimic, whereas KYSE450 and KYSE510 cell lines, which displayed high expression level of miR-503, were chosen to transfect the inhibitor. qPCR analysis showed that the expression of miR-503 was significantly increased by mimic transfection (*P* < 0.001, Figure1D) and significantly decreased by inhibitor transfection (*P* < 0.001, Figure1E).

**Figure 2:**
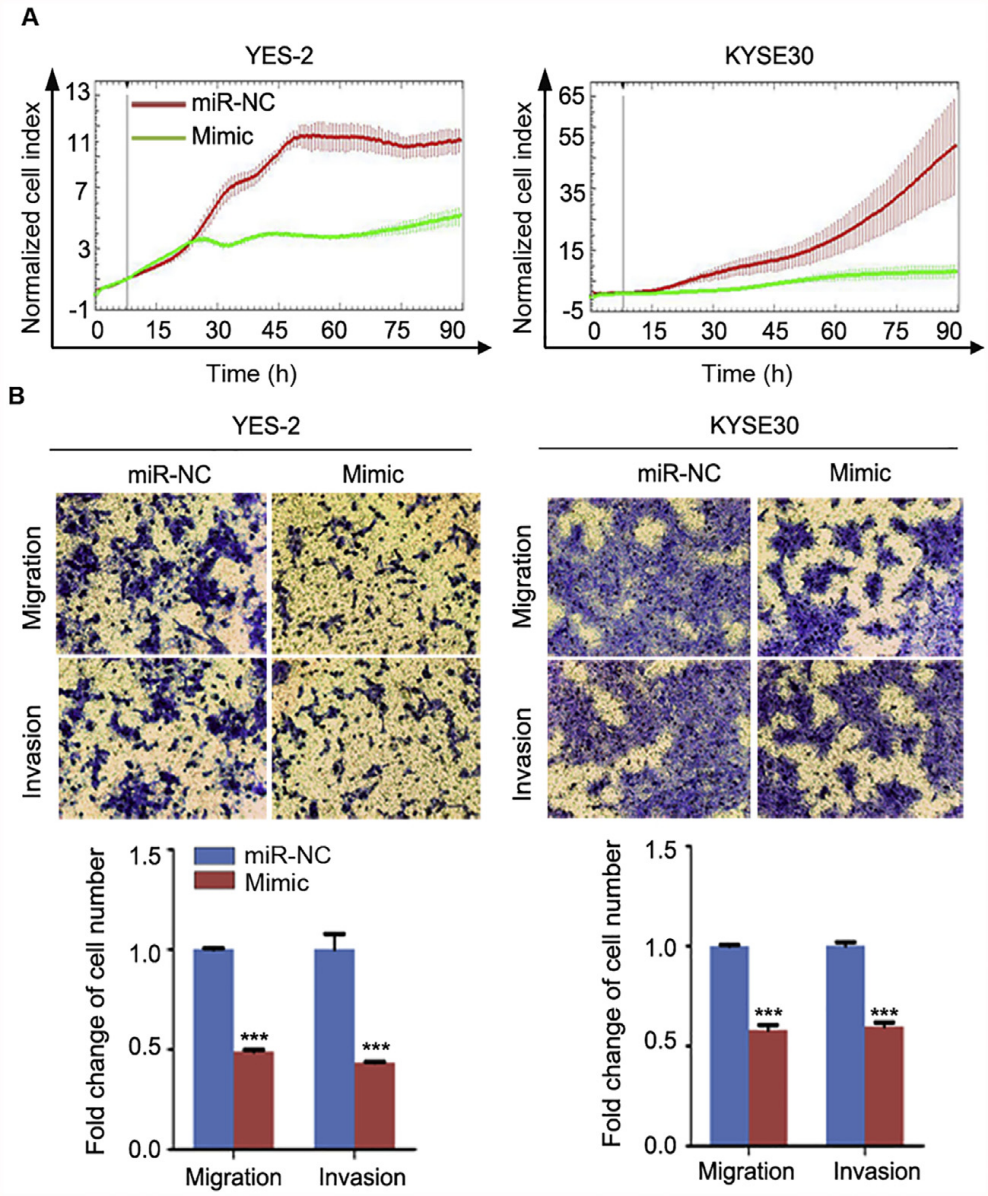
**Upregulation of miR-503 expression inhibits ESCC malignant phenotype** After YES-2 and KYSE30 cells were transfected with miR-503 mimic or miR-NC 24 h, their proliferation ability was detected by RTCA-MP system (**A**), whereas migration and invasion assays were performed using Transwell (**B**). Original magnification, 100×. Each bar represents the mean of three independent experiments. Data were analyzed using two-sided *t*-test. ^*^P < 0.05, ^**^P < 0.01, ^***^P < 0.001.

**Figure 3:**
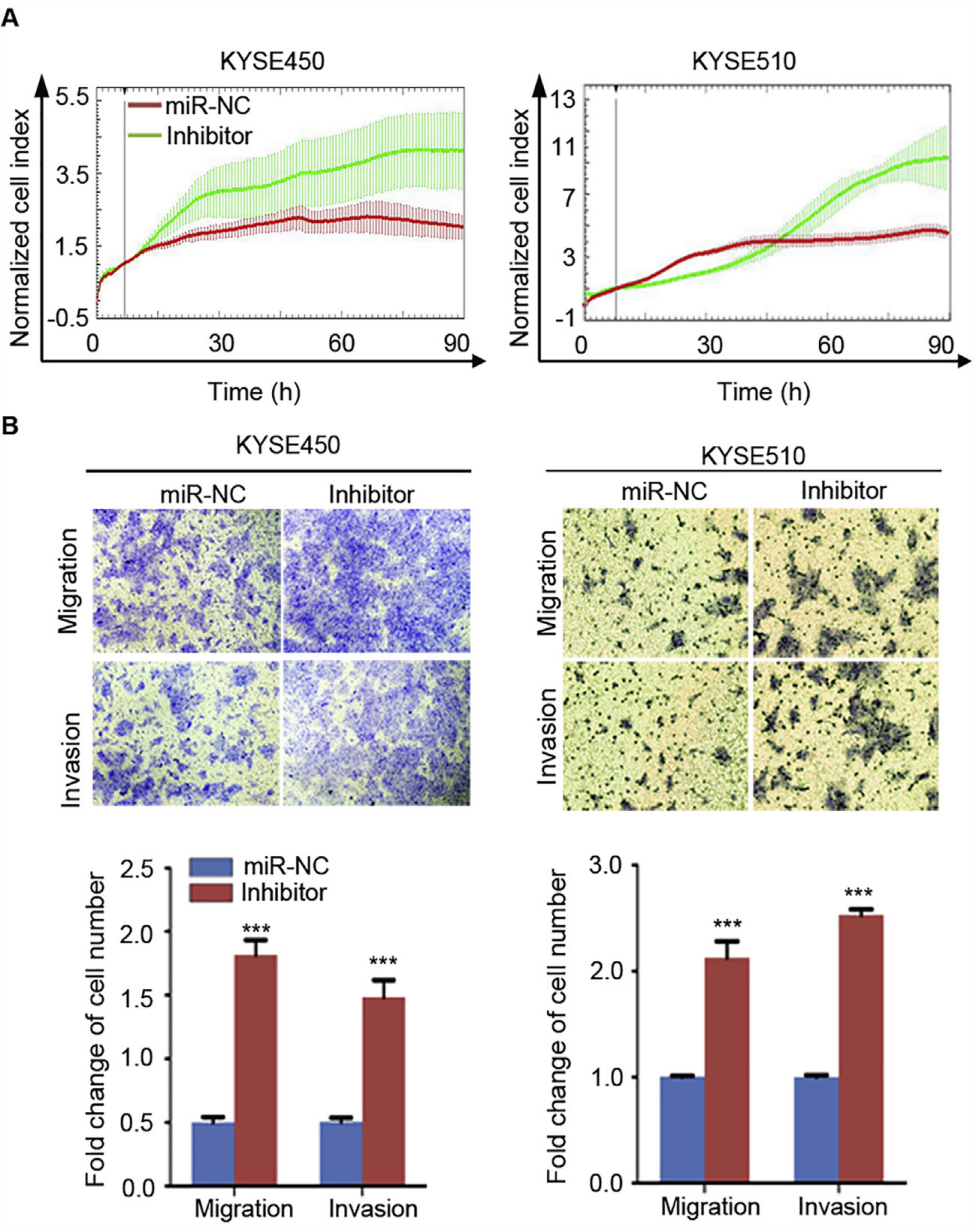
**Silencing of miR-503 expression promotes ESCC malignant phenotype** Cells were transfected with miR-503 inhibitor or miR-NC. **A.** The proliferation ability was detected by RTCA-MP system 24 h after transfection. **B.** Migration and invasion assays were performed with Transwell 24 h after transfection. Original magnification, 100×. Each bar represents the mean of three independent experiments and data were statistically analyzed by two-sided *t*-test. ^*^P < 0.05, ^**^P < 0.01, ^***^P < 0.001.

We next conducted cell phenotype assays to evaluate the effects of manipulating miR-503 expression on cell proliferation, migration, and invasion. Using the RTCA-MP system, we found that the proliferation ability of YES-2 and KYSE30 cells transfected with miR-503 mimic was markedly attenuated compared to the cells transfected with negative control (Figure2A). In addition, Transwell assay showed that migration and invasion abilities of YES-2 and KYSE30 cells transfected with miR-503 mimic were significantly decreased compared to the cells transfected with negative control (*P* < 0.001, Figure2B). Similar experiments were also performed in KYSE450 and KYSE510 cells transfected with the inhibitor of miR-503. As expected, silenced miR-503 expression with inhibitor in KYSE450 and KYSE510 increased cell proliferation, migration, and invasion ability, compared to the cells transfected with the control (Figure 3).

**Figure 4:**
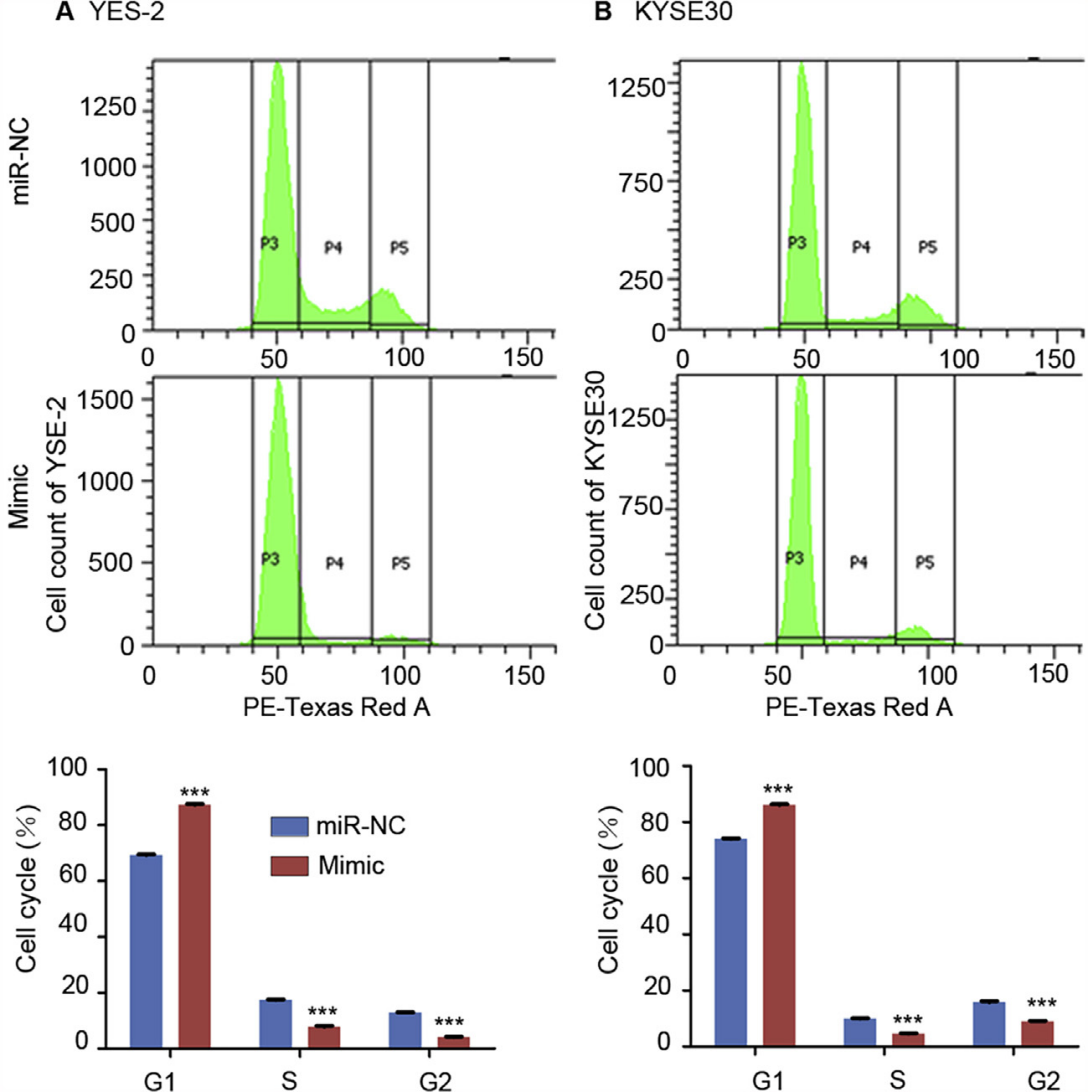
**Upregulation of miR-503 expression induces cell cycle G1/S arrest** ESCC cell lines YES-2 **A.** and KYSE30 **B.** were transiently transfected with miR-NC or miR-503 mimic. Flow cytometry was performed 24 h after transfection. The histograms of PI staining are presented in the upper panels. Percentage of cells at G1, S, and G2 phases of the cell cycles representing the average of triplicate experiments is presented in the bottom panels for quantification. ^*^P < 0.05, ^**^P < 0.01, ^***^P < 0.001.

Given the suppressive effect of miR-503 on cell proliferation, we speculate that miR-503 might affect cell cycle. We thus carried out flow cytometry for cell cycle analysis. As a result, we found a significant increase in the percentage at G1 phase and a significant decrease at S phase and G2 phase of the YES-2 cells transfected with miR-503 mimic, as compared to cells transfected with the negative control (*P* < 0.001, Figure4A). A similar pattern was also observed for KYSE30 cells (Figure4B). These results indicate that miR-503 could induce cell cycle G1/S arrest.

### miR-503 negatively regulates cyclin D1 expression

**Figure 5:**
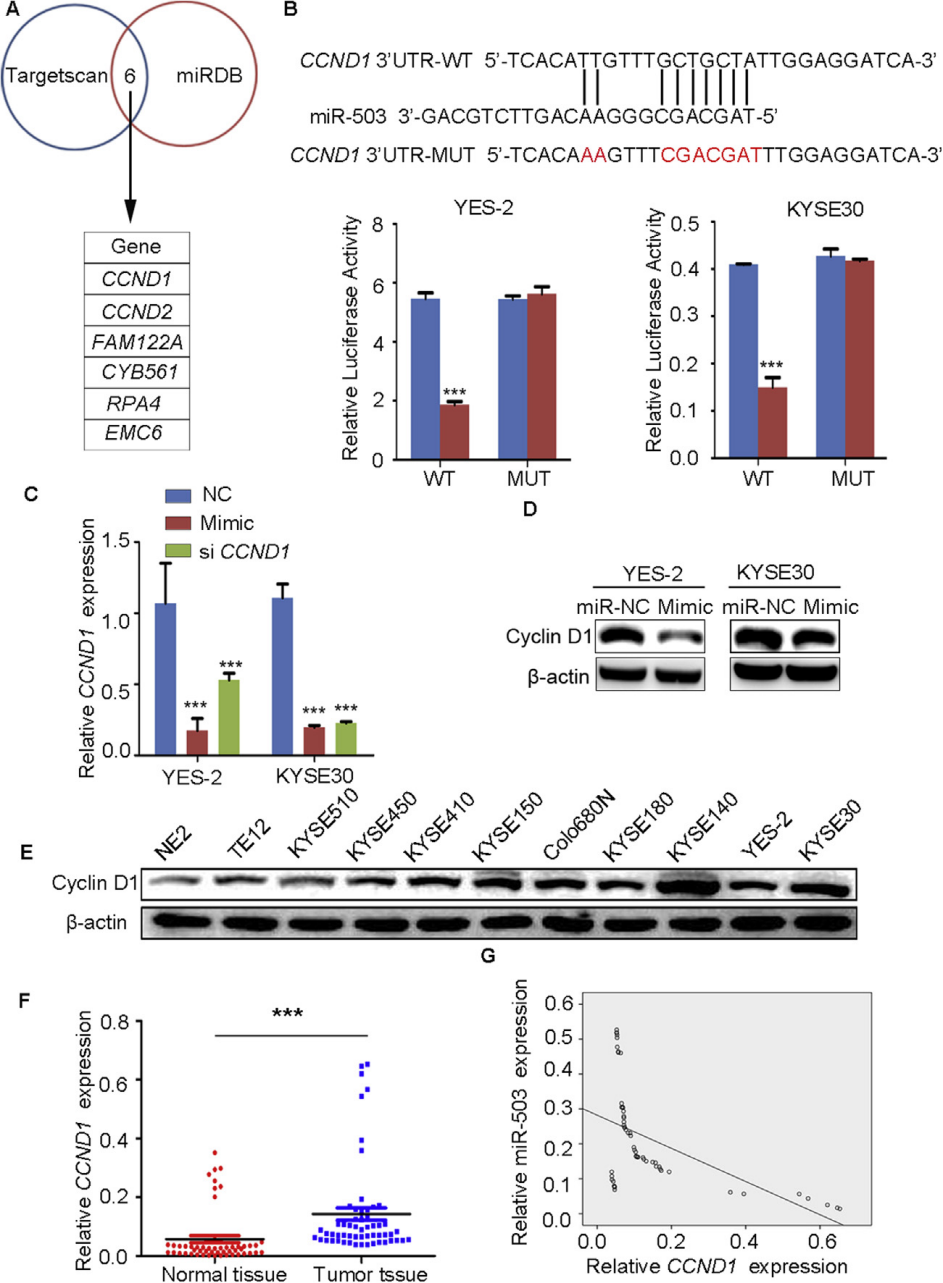
**miR-503 regulates the expression of *CCND1* by targeting its 3′UTR** **A.** Venn diagram and the list of putative miR-503 target genes commonly predicted by TargetScan and miRDB. **B.** Predicted miR-503 target sequence in 3′UTR of *CCND1* and the positions of mutated nucleotides (red). Relative luciferase activity of luciferase reporter plasmids containing the wild-type or mutant *CCND1* 3′UTR was determined in YES-2 and KYSE30 cells that were co-transfected with the miR-503 mimic or miR-NC. *Renilla* luciferase activity served as an internal control. **C.** qPCR analysis of *CCND1* expression in YES-2 and KYSE30 cells 48 h after transfected with miR-503 mimic or negative control. **D.** Western blotting analysis of cyclin D1 in YES-2 and KYSE30 cells 48 h after transfected with miR-503 mimic or negative control. **E.** Western blotting analysis of endogenous cyclin D1 expression in NE2 and ten ESCC cell lines. **F.** qPCR analysis of *CCND1* expression in 57 pairs of ESCC tissue samples. **G.** Correlation analysis of *CCND1* mRNA expression and miR-503 expression in the paired ESCC tumor samples from 57 patients. Data shown in panels B, C, and F were analyzed using Student’s *t*-test. Pearson correlation was used to calculate r and *P* values in panel G. ^*^P < 0.05, ^**^P < 0.01, ^***^P < 0.001.

The results above indicated that miR-503 might function as a tumor suppressor in ESCC. To further explore its molecular mechanisms, we firstly predicted the candidate target genes of miR-503 using TargetScan7.0 (http://www.targetscan.org) and miRDB (http://www.mirdb.org/miRDB/). As a result, 6 candidate genes that contain miR-503 binding sequence in their 3′UTRs were commonly found using these two bioinformatics methods (Figure5A). Among them, *CCND1* has been reported as oncogene before [16], [17], whereas the functions of the other five candidate target genes in tumor have not been defined yet. We thus selected *CCND1* for further investigation.

**Supplementary Figure 1:**
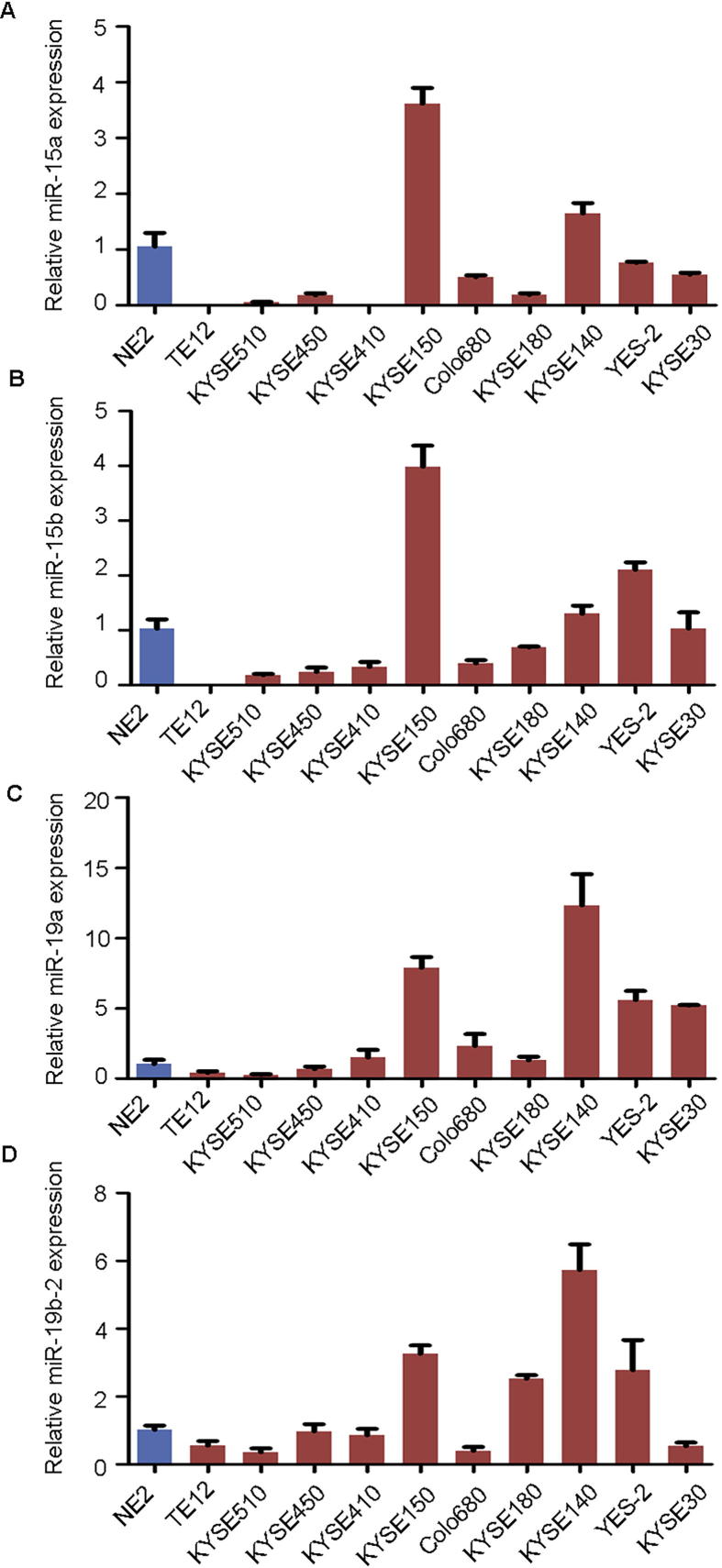
**Expression of miR-15 and miR-19 transcripts in NE2 and 10 ESCC cell lines**Expression of miR-15a **(A**), miR-15b (**B**), miR-19a (**C**), and miR-19b-2 (**D**) in NE2 and 10 ESCC cell lines was evaluated using qPCR and presented as fold change relative to the expression in NE2. All the experiments were performed at least three times.

According to TargetScan7.0, *CCND1* could be targeted by miR-15, miR-16, and miR-19 at different binding sites. Increased miR-16 expression has been reported in 40 ESCC tissues compared with their paired adjacent normal tissues and upregulation of miR-16 in ESCC cells could inhibit cell apoptosis and promote cell growth by regulating the protein expression of SOX6 and RECK [19]. The expression and function of miR-15 and miR-19 have not been reported before. miR-15 has two transcripts (miR-15a and miR-15b), and miR-19 has three transcripts (miR-19a, miR-19b-1, and miR-19b-2). We thus examined the expression of these miRNAs in NE2 and the 10 aforementioned ESCC cell lines (Figure S1). We detected expression of miR-15a, miR-15b, miR-19a, and, miR-19b-2 but not miR-19b-1 in the cell lines examined. However, none of miR-15a, miR-15b, miR-19a, miR-19b-2 showed consistent differential expression between NE2 and ESCC cells.

The complementary binding sites of miR-503 and *CCND1* 3′UTR (nt 1960–1966) are shown in Figure5B. To test whether *CCND1* is a direct target of miR-503, we generated a pair of plasmids, *i.e.*, pmiR-*CCND1*-WT plasmid, which contains *CCND1* 3′UTR sequence spanning the miR-503 binding site in the luciferase plasmid vector, and pmiR-*CCND1*-MUT plasmid, which contains the corresponding mutant counterpart (Figure5B). The luciferase construct, *Renilla* plasmid, and miR-503 mimic or negative control were cotransfected into YES-2 or KYSE30 cells. As shown in Figure5B, overexpression of miR-503 significantly reduced the luciferase activity of WT plasmid, whereas luciferase activity from mutant construct was not significantly changed. These data demonstrate a direct interaction of *CCND1* 3′UTR with miR-503.

We then looked at the effects of overexpression of miR-503 on the expression level of *CCND1* using si *CCND1* as a positive control. As shown in Figure5C, compared to the cells transfected with the negative control, expression of *CCND1* was significantly decreased in the YES-2 and KYSE30 cells transfected with miR-503 mimic or si *CCND1*. Western blotting analysis further indicated that cyclin D1 expression was reduced at protein level with the overexpression of miR-503 mimic (Figure5D).

### miR-503 expression is inversely correlated with *CCND1* expression in ESCC tissues

Protein expression of cyclin D1 in NE2 and the ten aforementioned ESCC cell lines was evaluated. Our results showed increased expression of cyclin D1 in the ESCC cell lines (Figure5E), which is opposite to the downregulation of miR-503 expression observed in ESCC cell lines (Figure1C).

We thus evaluated *CCND1* expression in the tumor tissues from ESCC patients. qPCR results revealed that *CCND1* expression was significantly upregulated in ESCC tissues compared to the adjacent non-tumorous samples (*P* < 0.001; Figure5F). Moreover, we plotted the relative expression of miR-503 against that of *CCND1* in the patients (Figure5G). Our data revealed a significant negative correlation between *CCND1* expression and miR-503 expression in ESCC tumor samples (*P* < 0.001, *r* = −0.523, Pearson).

### Overexpression of *CCND1* could partially diminish the tumor suppression of ESCC by miR-503

**Figure 6:**
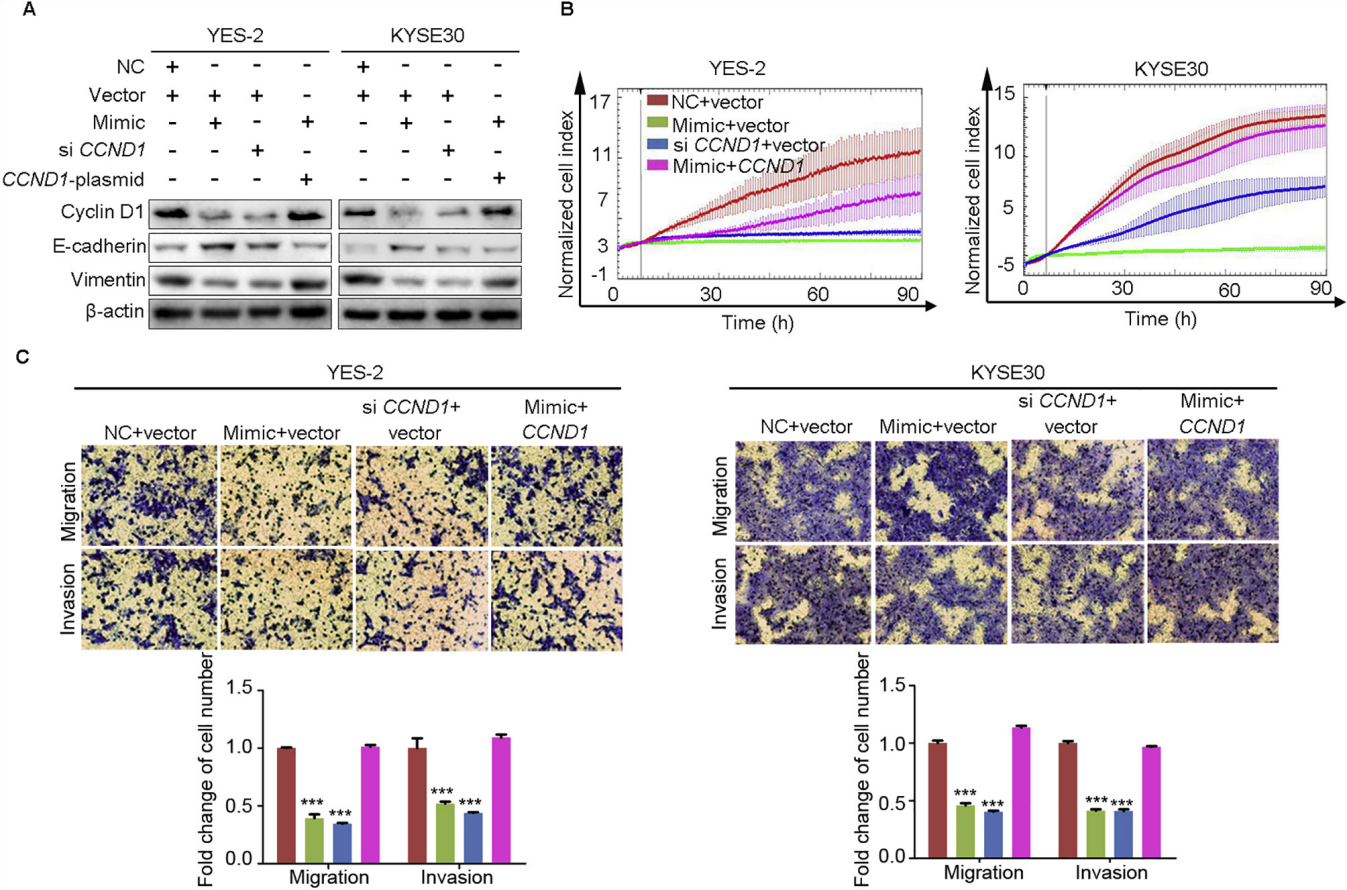
**Overexpression of *CCND1* could partly diminish the tumor suppressive effect of miR-503 on ESCC** YES-2 and KYSE30 cells were cotransfected with miR-503 mimic, *CCND1* siRNA or negative control and pcDNA3.1-vector or pcDNA3.1-*CCND1* and incubated for 24 h. **A.** Western blotting analysis of cyclin D1, vimentin, and E-cadherin using β-actin as a loading control. **B.** The proliferation ability of YES-2 and KYSE30 cells after various transfections was evaluated using RTCA-MP system. **C.** Migration and invasion assays of YES-2 and KYSE30 cells after various transfections were performed using Transwell. Original magnification, 100×. Each bar represents the mean of three independent experiments. Data were analyzed using the two-sided *t-*test. ^*^P < 0.05, ^**^P < 0.01, ^***^P < 0.001.

E-cadherin and vimentin are associated with cell motility and tumor metastasis. To explore the molecular mechanism of miR-503 in ESCC, we overexpressed miR-503 in YES-2 and KYSE30 cell lines in the presence or absence of *CCND1* overexpression. We found that overexpressing miR-503 led to the decreased protein expression of cyclin D1 and vimentin but an increased expression of E-cadherin. However, the altered expression of vimentin and E-cadherin can be partially reversed by overexpressing *CCND1* in YES-2 and KYSE30 cell lines (Figure6A).

To see whether cyclin D1 could rescue miR-503′s effect on ESCC, we silenced the endogenous *CCND1* expression by overexpression of miR-503 mimic and of *CCND1* in ESCC simultaneously. As shown in Figure6B, silenced expression of *CCND1* using si *CCND1* (si *CCND1* + vector) could mimic tumor suppressive effect of miR-503 mimic overexpression (mimic + vector) on ESCC cell proliferation, whereas overexpression of *CCND1* (mimic + *CCND1*) could partially diminish the tumor suppressive effect of miR-503 on ESCC cell lines. Similarly, knocking down expression of *CCND1* (si *CCND1* + vector) could mimic tumor suppressive effect of miR-503 mimic (mimic + vector) on ESCC cell migration and invasion, whereas overexpression of *CCND1* (mimic + *CCND1*) could partially reverse the tumor suppressive effect of miR-503 on ESCC cell lines (Figure6C).

## Discussion

Previous studies have shown that miR-503 expression is dysregulated in various physiological and pathological processes, including in human cancers. miR-503 plays a crucial role in tumorigenesis and cancer progression. It has been reported that miR-503 inhibits prostate cancer progression by repressing ZNF217 expression [20]. Additionally, miR-503 can also suppress hepatocellular carcinoma metastasis through Rho guanine nucleotide exchanger factor 19 [21].

In the present study, we demonstrated, for the first time, the tumor suppressive role of miR-503 in ESCC. Examination of miR-503 expression in paired clinical tumor samples of ESCC patients showed that miR-503 expression was downregulated in most ESCC tissue samples relative to adjacent normal tissues. Functional studies by manipulating miR-503 expression in different ESCC cell lines further showed that overexpression of miR-503 inhibited cell proliferation, migration, and invasion, whereas suppression of miR-503 expression had the opposite effects. These data support miR-503 as a tumor suppressor.

We also symmetrically examined miR-503 expression in 10 ESCC cell lines and revealed a general trend of decreased miR-503 expression in ESCC cells compared to the immortalized esophageal epithelial cell line NE2. We further showed that enhanced expression of miR-503 suppressed cell proliferation, migration, and invasion in two ESCC cell lines examined. Our observation is different from the observation reported in another ESCC cell line EC9706 with increased miR-503 expression, showing the tumor suppressive effects of the decreased miR-503 expression [18]. Such discrepancy could be due to cell specificity of the cell lines used, since ESCC is a complex disease and each ESCC cell line may have different origin and thus different characteristics.

miRNAs function by inhibiting the expression of target genes. Based on our data in the present study, the function of miR-503 target genes in ESCC might be associated with proliferation and metastasis. Overexpression of cyclin D1 is frequently observed in a variety of tumors, especially in ESCC [22], [23], [24]. There are many factors contributing to the overexpression of cyclin D1 in cancer, such as amplification and abnormal transcription of *CCND1* [22], [23], [24], [25], [26]. As a key mediator of cell cycle progression, cyclin D1 forms complex with cyclin-dependent kinase 4 (CDK4) and CDK6 to inactivate the retinoblastoma (RB) pathway via hyperphosphorylation of the RB protein and result in acceleration of E2F-mediated gene transcription [27]. Blockage of cyclin D1 expression could induce cell cycle G1/S phase arrest, thus leading to the inhibition of cell proliferation [25], [26]. Interestingly, our study shows that overexpression of miR-503 in ESCC cell lines also induced G1/S arrest. In agreement with this observation, we found that cyclin D1 is the direct target of miR-503 by dual-luciferase reporter assay and western blot. Therefore, miR-503 could inhibit ESCC cell proliferation by targeting *CCND1* and inducing cell cycle G1/S arrest.

Cyclin D1 is also reported to be involved in the regulation of tumor metastasis [28], [29], [30]. *CCND1*-deficient mice exhibit the tumor resistance because of the reduced motility and invasiveness of *CCND1*−/− tumor-associated macrophages [28], [29]. Decreased cyclin D1 and cyclin D1-CDK4/6 kinase activity reduces invasion and migration in breast cancer cells [30]. Cyclin D1/P21 regulates breast cancer cell migration and invasion through TGFβ pathway [31], [32]. Cyclin D1 stimulated EMT factors (including E-cadherin, N-cadherin, and vimentin) through PI3K/AKT pathway [33]. In this study, we found that overexpression of miR-503 reduced the protein levels of cyclin D1 and vimentin, but increased the expression of E-cadherin, which can be partially revised by overexpression of *CCND1*. Moreover, silenced expression of *CCND1* could recapitulate the tumor suppressive effect of miR-503 mimic on ESCC, whereas overexpression of *CCND1* could partially reverse the tumor suppressive effect of miR-503 mimic on ESCC cells. Therefore, miR-503 could inhibit ESCC cell migration and invasion by targeting *CCND1* through decreasing vimentin level and increasing E-cadherin level.

In conclusion, our study demonstrates that miR-503 has a tumor suppressor role in ESCC cell lines and tumor samples by negatively regulating the expression of its direct target, *CCND1*, in ESCC.

## Materials and methods

### Clinical tissue and samples

71 pairs of ESCC tumor tissues and the normal adjacent counterparts were collected from ESCC patients at the Department of Thoracic Surgery of Cancer Hospital, Chinese Academy of Medical Sciences and Peking Union Medical College from 2003 to 2012. All patients have provided the written consent. The clinical information of patients included in this study is presented in Table 1.

### Cell culture

Ten human ESCC cell lines were examined in this study, which were kindly provided by Professor Yutaka Shimada from Kyoto University, Japan. These include TE12, KYSE510, KYSE450, KYSE410, Colo680N, KYSE180, KYSE140, YES-2, KYSE150, and KYSE30. The cells were maintained in RPIM-1640 medium supplemented with 10% fetal bovine serum (FBS) and antibiotics. The immortalized esophageal epithelial cell line NE2 was provided by Professor Enmin Li from Shantou University. These cells are cultured in a 1:1 mixture of EpiLife and dKSFM (Gibco) as described previously [3].

### Cell transfection

miR-503 mimic, inhibitor, and siRNA for *CCND1* were purchased from RiboBi (Guangzhou, China) for transient transfection. Transfection was performed at the final concentration of 25 nM for mimic, 75 nM for inhibitor, 50 nM for si *CCND1* with Lipofectamine 3000 (Invitrogen, Carlsbad, California) as instructed by the manufacturer.

### RNA extraction and real-time PCR

RNA was extracted using TRIzol Reagent (Invitrogen). 2 µg total RNA was used for cDNA synthesis with Superscript II reverse transcriptase (Invitrogen) according to the manufacturer’s instruction. qPCR was conducted to determine the expression levels of hsa-miR-503 and *CCND1* mRNA using Applied Biosystems 7300 Real-Time PCR System, with the U6 small nuclear RNA and *GAPDH* as internal controls, respectively. The sequences of the primers used are listed in Table S1.

qPCR data were quantified according to the 2^−ΔCT^ method, and data analysis was performed and visualized using GraphPad Prism v.5.

### Cell proliferation assay

Cell proliferation assay was performed using RTCA-MP system as described previously [12], [34].

### Migration and invasion assays

Migration and invasion assays were performed with Transwell insert chambers (Corning, New York, NY) as described previously [12] with a density of 2 × 10^5^ ESCC cells were seeded in the upper chambers of Transwell and incubated for 24 h. Cells were fixed in methanol for 5 min and stained with crystal violet for 5 min. The migrated cells were examined under a phase-contrast microscope for imaging analysis. Cells in nine random microscopic fields (100 × magnification) were counted per well. For the invasion assay, the upper chamber membrane was pre-coated with 100 μl of 2% Matrigel (BD Biosciences, San Jose, CA) and similar steps were performed as for the migration assay.

### Luciferase reporter assay

The *CCND1* 3′UTR wild type fragment (WT) and mutant fragment (MUT) were separately inserted in the firefly luciferase gene downstream in the pmirGLO (Generay, Shanghai, China) plasmid. Cells were cotransfected with WT or MUT plasmid and miRNA negative control or miR-503 mimic using Lipofectamine 3000 (Invitrogen). 36 h after transfection, cell lysates were collected for measurement of the luciferase activities (normalized to luciferase activity of the *Renilla*) using Dual-Luciferase Reporter Assay System (Promega, Madison, WI) according to the manufacturer’s instruction.

### Western blotting analysis

Western blot analysis was performed as described previously [12]. Primary antibodies used in this study include anti-β-actin, anti-vimentin, anti-cyclin D1, and anti-E-cadherin (Santa Cruz Biotechnology, Santa Cruz, CA). The horseradish peroxidase-conjugated secondary antibodies used include goat-anti-mouse IgG (1:2000) and goat-anti-rabbit IgG (1:3000). After incubation with chemiluminescence substrate, images of protein bands were taken by Image Reader LAS-4000 (Fuji Ltd, Osaka, Japan) and then analyzed using Multi Gauge V3.2 software.

### Flow cytometric analysis

Cells were fixed in 100% methanol for 30 min at −20 °C, and then resuspended in 300 μl propidium iodide (PI) staining solution (0.5 mg/ml PI). Cell-cycle progression was analyzed with a LSRII flow cytometer (BD Biosciences) and Modifit3.1 Crack analysis software.

### Statistical analysis

All the data were analyzed using SPSS software (version 17.0). Differences between groups were analyzed using the Student’s *t*-test (two-tailed). Association between miR-503 expression and various clinicopathological parameters was determined using a Pearson’s chi-squared (*χ*^2^) test [13]. Correlation Pearson analysis was performed to evaluate the association between miR-503 expression and *CCND1* expression and calculate coefficient (*r*) and *P* values. ^∗^*P* < 0.05, ^∗∗^*P* < 0.01, ^∗∗∗^*P* < 0.001 versus control.

## Authors’ contributions

YS designed the study. LJ conducted the experiments. ZZ, LZ, and LX collected the ESCC tissues and performed the pathological analysis. LJ, ZZ, YS, and QZ wrote the manuscript. All authors had read, edited, and approved the final manuscript.

## Competing interests

The authors have declared no competing interests.

## Supplementary material

Supplementary Table S1 **List of primers used in current study**
