# Supplementary material for: Downregulation of miR-503 Promotes ESCC Cell Proliferation, Migration, and Invasion by Targeting Cyclin D1
Source: Genomics Proteomics Bioinformatics. 2017 Jun 9;15(3):208–17. doi: 10.1016/j.gpb.2017.04.003 (PMC5487524; doi:10.1016/j.gpb.2017.04.003)
Supplement: Supplementary Table S1 — List of primers used in current study [file mmc1.docx]

**Table S1 List of primers used in current study**

| **Gene** | **Primer** | **Primer sequence (5′–3′)** |
| --- | --- | --- |
| *MIR503* | RT | GTCGTATCCAGTGCAGGGTCCGAGGTATTCGCACTGGATACGACCTGCAG |
|  | Forward | TAGCAGCGGGAACAGTTCTGCAG |
|  | Reverse | GTGCAGGGTCCGAGGT |
| *MIR15A* | RT | GTCGTATCCAGTGCAGGGTCCGAGGTATTCGCACTGGATACGACCACAAA |
|  | Forward | TAGCAGCACATAATGGTTTGTG |
|  | Reverse | GTGCAGGGTCCGAGGT |
| *MIR15B* | RT | GTCGTATCCAGTGCAGGGTCCGAGGTATTCGCACTGGATACGACTGTAAA |
|  | Forward | TAGCAGCACATCATGGTTTACA |
|  | Reverse | GTGCAGGGTCCGAGGT |
| *MIR19A* | RT | GTCGTATCCAGTGCAGGGTCCGAGGTATTCGCACTGGATACGACTGTAGT |
|  | Forward | AGTTTTGCATAGTTGCACTACA |
|  | Reverse | GTGCAGGGTCCGAGGT |
| *MIR19B1* | RT | GTCGTATCCAGTGCAGGGTCCGAGGTATTCGCACTGGATACGACGCTGGA |
|  | Forward | AGTTTTGCAGGTTTGCATCCAGC |
|  | Reverse | GTGCAGGGTCCGAGGT |
| *MIR19B2* | RT | GTCGTATCCAGTGCAGGGTCCGAGGTATTCGCACTGGATACGACTGAAAT |
|  | Forward | AGTTTTGCAGGTTTGCATTTCA |
|  | Reverse | GTGCAGGGTCCGAGGT |
| *U6* | Forward | CTCGCTTCGGCAGCACA |
|  | Reverse | AACGCTTCACGAATTTGCGT |
| *CCND1* | Forward | CCTCGGTGTCCTACTTCA |
|  | Reverse | CTCCTCGCACTTCTGTTC |
| *GAPDH* | Forward | TATGACAACAGCCTCAAGAT |
|  | Reverse | AGTCCTTCCACGATACCA |
